# Obesity and the Brain

**DOI:** 10.3390/ijms23116145

**Published:** 2022-05-30

**Authors:** Jacek Karczewski, Aleksandra Zielińska, Rafał Staszewski, Piotr Eder, Agnieszka Dobrowolska, Eliana B. Souto

**Affiliations:** 1 Department of Environmental Medicine, Poznan University of Medical Sciences, Fredry 10, 61-701 Poznan, Poland; 2 Department of Gastroenterology, Dietetics and Internal Diseases, Poznan University of Medical Sciences, Fredry 10, 61-701 Poznan, Poland; piotreder@ump.edu.pl (P.E.); dobrowolska.agnieszka@spsk2.pl (A.D.); 3 Institute of Human Genetics, Polish Academy of Science, Strzeszynska 32, 60-479 Poznan, Poland; aleksandra.zielinska@igcz.poznan.pl; 4 Hypertension Angiology and Internal Medicine, Poznan University of Medical Sciences, Fredry 10, 61-701 Poznan, Poland; rafal.staszewski@ump.edu.pl; 5 Department of Pharmaceutical Technology, Faculty of Pharmacy, University of Porto, Rua de Jorge Viterbo Ferreira, No. 228, 4050-313 Porto, Portugal; ebsouto@ff.up.pt; 6 REQUIMTE/UCIBIO, Faculty of Pharmacy, University of Porto, Rua de Jorge Viterbo Ferreira, No. 228, 4050-313 Porto, Portugal

**Keywords:** obesity, neuropsychiatric diseases, neuroinflammation, microbiota, dysbiosis

## Abstract

Innate and adaptive immunity are essential for neurodevelopment and central nervous system (CNS) homeostasis; however, the fragile equilibrium between immune and brain cells can be disturbed by any immune dysregulation and cause detrimental effects. Accumulating evidence indicates that, despite the blood–brain barrier (BBB), overactivation of the immune system leads to brain vulnerability that increases the risk of neuropsychiatric disorders, particularly upon subsequent exposure later in life. Disruption of microglial function in later life can be triggered by various environmental and psychological factors, including obesity-driven chronic low-grade inflammation and gut dysbiosis. Increased visceral adiposity has been recognized as an important risk factor for multiple neuropsychiatric conditions. The review aims to present our current understanding of the topic.

## 1. Introduction

Interactions between the immune and nervous system are gaining attention across multiple neuropsychiatric diseases [1]. The classic assertion that the central nervous system (CNS) is an immune privileged organ has undergone a radical paradigm shift. Although protected by specialized physical barriers, the brain is not immunologically isolated from the peripheral immune system. It seems the interactions between neurons, microglia, and the immune system contribute to the functional properties of a brain, such as cognition, learning performance, and social behavior.

Accumulating data suggest the complex interactions between neurons and microglia, the brain parenchyma-resident macrophages that account for approximately 10% of CNS cells [2]. It seems that microglial activity synchronizes with the development, maturation and senescent of the CNS during the lifespan via the adoption of different regulatory networks [2]. The primary function of microglia covers the surveillance of baseline neuronal activity and rapid response to tissue damage [3]. Recently, it has been recognized that microglia also contribute to essential neurodevelopmental processes, including angiogenesis, phagocytosis, and synaptic pruning [4]. As microglia exhibit the ability to secrete both growth and noxious factors, and phagocyte neural progenitors, they control the populations of other resident CNS cell types. Microglia can directly induce cell death using reactive oxygen species (ROS) and nerve growth factor (NGF) [4]. They can also promote neuron survival by secreting trophic factors and cytokines [5,6]. Their ability to support astrocyte and oligodendrocyte differentiation has been demonstrated in vitro [7]. In turn, astrocytes as the most numerous glial cells in the CNS can play significant roles in promoting the blood–brain barrier (BBB) integrity and immunoregulation, release transforming growth factor-β (TGF-β), which modifies chromatin in microglia to limit their activation [8]. Astrocyte-derived TGF-β and interleukin (IL)-33 promote synaptic pruning by microglia, suggesting that neuroinflammation can result in the abnormal elimination of neuronal synapses and alterations in brain circuits [9]. Microglia can phagocyte living and apoptotic cells, debris, myelin, and synapses during neurodevelopment [4]. In addition, microglia can directly promote myelination via various secretory factors, including insulin growth-like factor-1 (IGF1) [7]. In the healthy adult brain, microglia maintain the homeostasis of synaptic circuits in the CNS [10]. Due to the expression of surface receptors for various neurotransmitters and purines, microglia can sense local neuronal activity [11]. In response, microglia can directly contact neurons via different processes [10] or can indirectly modulate neuronal firing rate via release of extracellular vesicles [12] and signaling molecules, including tumor necrosis factor (TNF)-α [13]. Thus, microglia are involved in activity-induced synaptic plasticity, for instance, as a part of motor learning and memory. During systemic inflammation, microglia can be activated and produce pro-inflammatory mediators and induce phagocytosis [14].

In contrast to resident microglia, which comprise the innate CNS immune cells compartment, the presence of adaptive immune cells in the brain had long been regarded as pathologic. However, in healthy subjects, dendritic cells, macrophages, innate lymphoid cells (ILC)2, B cells, and T cells that reside in the meninges, the choroid plexus, and the cerebrospinal fluid monitor the brain’s borders, providing additional protection to the BBB [15]. It seems that the choroid plexus participates not only in transmigration, but also in the activation of T cells in response to peripheral inflammatory signals [16]. Although T cells do not generally penetrate the parenchyma under non-inflammatory conditions, they can secrete pro-inflammatory mediators that influence CNS function [17,18].

Preclinical studies demonstrate that adaptive immunity promotes cognitive performance: mice with severe combined immune deficiency exhibit altered spatial learning and memory, although those symptoms are reversed by transferring exogenous T cells [19]. Similar findings have been reported on social behavior [17]. From a neurobiological perspective, levels of neurogenesis in adult transgenic mice that overexpress a CNS-specific T cell receptor are higher than those in mice that overexpress a T cell receptor for non-CNS-specific antigens [20]. The beneficial effects of self-reactive T cells on the maintenance of neuronal function have led to the concept of protective autoimmunity, in which adaptive immune cell function maintains tissue function. A protective role of T helper 2 (Th2) cells in CNS damage for IL-4 signaling in neurons in multiple sclerosis (MS) models is another example of the positive effect of adaptive immunity in the CNS [21,22]. Lack of B cells has also been found not to impair learning behavior in mice [23]. The interactions between the CNS and the periphery are mediated by two drainage systems: the glymphatic system which provides nutrients for brain cells and clearance of extracellular metabolites, as well as the meningeal lymphatic system which allows drainage from the CNS to cervical lymph nodes and provides the connection to the peripheral immune system [24]. Via the latter system, molecules and immune cells from the CNS can be transported to lymphoid organs and initiate an adaptive immune response [25].

This review discusses the most recent studies of the influence of obesity on a broad spectrum of different disorders, mainly metabolic syndrome, diabetes, systemic hypertension, heart failure, or kidney failure. Most recent studies have shown that the roles of overnutrition can induce hypothalamic inflammation in neurodegeneration. These studies have provided consistent evidence of smaller cortical thickness or reduction in the gray matter volume in people with overweight and obesity. Moreover, adipocytes, macrophages of the adipose tissue, and gut dysbiosis can result in the secretion of the cytokines and chemokines, crossing the blood–brain barrier. They also can induce microglia, which release proinflammatory cytokines. Consequently, it can lead to chronic low-grade neuroinflammation and may be a key factor for apoptotic signaling and neuronal death [26]. Furthermore, this work mentions a poor cognitive performance, mainly in executive functions, in individuals with obesity. It is worth to underline that the neuroinflammatory and neurodegenerative mechanisms are related to obesity [27]. Therefore, there is urgent need to develop the efficient prevention and treatment strategies for obese patients.

## 2. Inflammation and Cognitive Performance

Mounting evidence indicates that both innate and adaptive immunities are essential for CNS homeostasis. Therefore, any immune dysregulation could disrupt the fragile equilibrium between immune cells, neurons, and microglia, resulting in a spectrum of neuropsychiatric diseases.

### 2.1. Infection-Related Inflammation

Evidence indicates that infection-related inflammation can substantially alter cognitive function. For instance, the data from a Danish longitudinal study shows that prior hospitalization that involved infection increased the risk of a major mood disorder (MMD) by 60%, while hospital contact with infection occurred in nearly 24% of individuals before a MMD [28]. Bacterial or viral infections during pregnancy have been linked to autism spectrum disorder (ASD) in children [29]. Similarly, an infection in a rodent fetus can lead to long-term cognitive alterations, including learning, memory, and attention impairments, which might explain why early infections increase the risk of psychosis in young adults [30,31]. In children, throat infections with Streptococcus pyogenes have been linked to a broad spectrum of neuropsychiatric disorders (possibly caused by the cross-reactivity of antibodies) [32,33]. Severe infections, such as herpes simplex virus (HSV) encephalitis, at any time during the lifespan, have been associated with long-term impairment of memory and brain atrophy [34]. Available data also suggests that infections with Toxoplasma gondii, CMV, and HSV in subjects with schizophrenia or bipolar disorder (BD) additionally affect the cognitive dysfunction, in particular a working memory [35]. Accumulating evidence regarding the impact of SARS-CoV-2 infections during the ongoing COVID-19 pandemic also indicates a strong cognitive dysfunction and mental health problems associated with inflammation after recovery, despite the age group [36,37,38]. COVID-19 disease has been characterized as a cytokine release syndrome in which elevated serum levels of pro-inflammatory cytokines correlate in a dose-response manner with respiratory failure, adverse respiratory distress syndrome, and other clinical outcomes [39]. The data shows that psychiatric symptoms and cognitive impairment can develop and persist months after infections (“long covid” condition).

Another compelling example includes sickness behavior (SB), characterized by a reduction in motor activity, motivation, social interactions and appetite, impairment of learning and memory, and often accompanied by fever [40]. This unspecific set of behavioral symptoms is highly conserved among species and provokes the immune response to maximize the metabolic resources for fighting the pathogen and promoting recovery and tissue repair [41]. Apparently, in infection-driven or systemic inflammation, the release of inflammatory mediators, notably TNF-α, IL-1β, IL-6, and IL-17A outside the CNS influences the brain via neural, mainly vagal pathways and interactions with surface receptors on cerebral endothelial cells and/or microglia, leading to leukocyte recruitment and impaired neurogenesis that promote SB [40]. For instance, a potent pro-inflammatory IL-1β has been demonstrated to repress long-term potentiation (LTP), an essential process for memory formation in the hippocampus, both in vitro [42,43] and in vivo in mice [44]. Reduced LTP has been correlated with the activity of stress-activated kinases and ROS in the hippocampal tissue. The potential mechanisms underlying the infection-related alterations in cognitive functions involve a molecular mimicry initiating chronic inflammation (autoimmune processes), and possibly a stress-induced potentiation of microglial inflammasome activation resulting in increased production of pro-inflammatory mediators [45,46]. Therefore, both innate and adaptive immune immunity could contribute to pathology. Infectious agents might also modulate the innate immune cells via epigenetic modifications, increasing the risk of neurodegenerative disorders [47]. Pathogens can also contribute directly to neurodegeneration, which leads to cognitive decline [48,49]. Altogether, it seems that due to the fragile equilibrium of the immune system and brain function, infections can lead to neuropsychiatric diseases. Infections can also promote autoimmune processes that contribute to those pathologies.

### 2.2. Autoimmunity

As mentioned, pathogen infections can involve molecular mimicry, triggering the autoimmune processes [32,50]. Neurological autoimmune diseases, such as neuromyelitis optica and autoimmune encephalomyelitis, can be driven by autoaggressive lymphocytes or anti-neuronal/anti-glial autoantibodies. MS is the classical and most common autoimmune encephalomyelitis, leading to demyelination and progressive degeneration. In early MS, the majority of subjects experience various transient physical deficits, including optic neuritis, hemiparesis, or sensory disturbances, in a relapsing-remitting presentation, often accompanied by cognitive decline and depression [51,52]. The most common cognitive deficits found in 40–60% of MS subjects include lower processing speed, impaired memory, and executive function, which can be improved to some extent with immunomodulatory therapy [53]. Data shows that cognitive impairment at the time of MS diagnosis predicts disability progression, transition to secondary progressive MS, and cortical thinning [54]. Furthermore, the risk of cognitive decline correlates with the progression of the disease and is highest in secondary progressive MS [55]. Inflammation in MS is believed to be primarily driven by autoreactive T cells, leading to microglial activation and myelin and neuronal damage. Evidence suggests that myelin-reactive Th17 and Th1 cells promoted by gut microbiota play a major role in migrating to the CNS, altering the BBB permeability, and inducing the migration of other leukocytes to the site [56,57]. Although bacterial metabolites such as polysaccharide A and short-chain fatty acids (SCFAs) can induce regulatory T (Treg) cells, the specific microbial factors responsible for pathogenic T cells activation remain unknown [57]. The numerical/functional deficits in the Treg cells subpopulation have also been suggested to play an important role in MS immunopathology [58,59], as well as B cells, which is supported by the positive effects of B cell-depleting therapies [60]. Relapses in MS subjects are affected by stressful events and infections, most frequently those of the upper respiratory tract [61]. Although a specific autoantigen in MS has not been identified yet, autoreactive T cells are assumed to target proteins of the myelin sheath, causing demyelination and, thereby, white matter damage [57,59,62]. Demyelination deprives neurons of protective factors and has been described to decrease axonal transport and synaptic density in demyelinated hippocampi [63]. The direct recognition of neurons by autoreactive T cells has also been suggested [64]. Grey matter damage, measured as the cortical lesion load or cortical thinning, is a good predictor of disease progression and cognitive decline [65]. The presence of gadolinium-enhancing lesions on brain MRI is associated with impaired cognitive performance even in stable MS subjects [66]. Although rare, psychotic symptoms such as schizophrenia can also occur in MS, and the overlapping genes are immune-related [67]. MS and various psychiatric disorders are accompanied by higher-order network impairments [1].

Another example includes psychosis occurring due to the autoantibodies against neutrophils, developed spontaneously in neoplastic diseases or after viral infections [68]. The autoantibodies can be directed against some intracellular antigens, including onconeural proteins or glutamic acid decarboxylase (GAD65). Although the occurrence of such antibodies is diagnostically useful, it is considered an epiphenomenon, since neuronal damage associated with neuropsychiatric symptoms is caused by autoreactive T cells [69]. Nonetheless, the pathogenic autoantibodies could be directed against other unspecified surface antigens involved in synaptic transmission and plasticity. Subjects with psychosis often manifest the anxiety, sleep disorders, mania, paranoia, memory impairment and disintegration of language, followed by a phase in which agitation and catatonia alternate, accompanied by abnormal movements and autonomic instability.

Interestingly, recent data indicates that autoimmune processes within the CNS induce a broad migration of intestinal IgA+ plasma cells differentiated from B cells stimulated with intestinal antigens, into the brain and spinal cord fluid to attenuate neuroinflammation in an IL-10-dependent manner [70]. The precise signals responsible for IgA+ plasma cells recruitment to the brain have not been determined yet, but the presence of cells in the brain has been shown at homeostasis, where they localize in proximity to dural venous sinuses to protect the CNS from the entry of pathogens [71]. These observations might lead to the development of novel therapeutic strategies for neuroimmune disorders based on IgA+ B cells promoting immunosuppression [70,71].

### 2.3. Chronic Low-Grade Inflammation

In contrast to transient inflammation, chronic low-grade inflammation can occur and cause neurotoxicity and neurodegeneration [72]. The pro-inflammatory cytokines released can have neurotoxic effects, leading to synaptic dysfunction, neuronal death, and inhibition of neurogenesis [73]. Circulating inflammatory mediators can disrupt epithelial tight junctions and compromise both the gut-vascular barrier (GVB) and the BBB integrity, providing a gateway for intestinal lumen-derived factors to reach the brain parenchyma, stimulating local immune cells, and start neuroinflammation [74]. For instance, higher systemic concentrations of LPS have been associated with microglial activation, neuronal cell death, cognitive impairment, and already mentioned cytokine-mediated SB [75]. IL-1β induces synaptic loss by promoting prostaglandin E2 production, leading to presynaptic glutamate release and postsynaptic N-methyl-D-aspartate (NMDA) glutamate receptor activation [76], and TNF-α causes neuronal death by activating TNF receptor 1 (TNFR1) and recruiting caspase 8 when the nuclear factor-κB (NF-κB) pathway is inhibited [77]. In addition, the complement system can be activated, promoting the phagocytic function of microglia, which might result in altered pruning of synapses [78]. Chronic, sterile low-grade inflammation occurs for instance during human aging (“inflammaging”) and can contribute to age-related diseases [79]. Aging-related cognitive impairment has been linked to both a chronic low-grade enteric and systemic inflammation, manifesting as an increase of microglia, T cells, and border-associated macrophages (BAM) in the CNS as well as gut dysbiosis [80,81]. Aging-associated B cells also migrate from the periphery to the meninges and differentiate into IgM+ plasma cells [81]. Importantly, gut dysbiosis promotes intestinal permeability that precedes the immune alterations in the CNS and induces symptoms of neuroinflammation, indicating that dysbiosis favors humoral signaling across the gut–brain axis [81]. Chronic low-grade inflammation has been also implicated in schizophrenia and other psychiatric disorders [82], and both systemic inflammation and acute infections have been associated with an increased cognitive impairment and exacerbation of symptoms in AD subjects [83].

**Figure 1:**
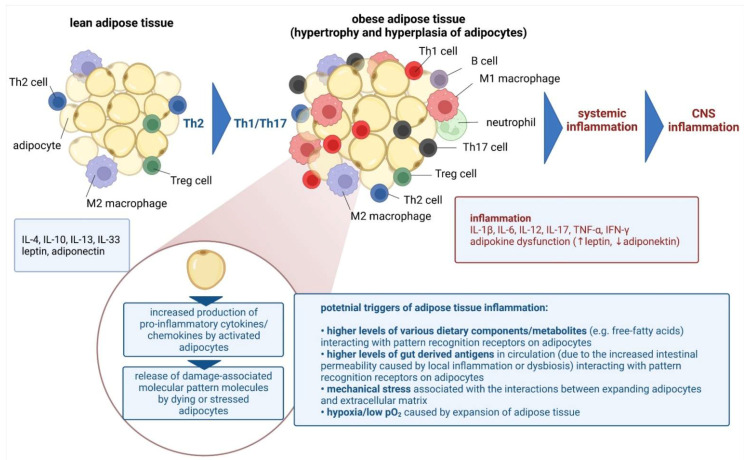
Chronic low-grade systemic inflammation in obesity resulting in the central nervous system inflammation [own drawing]. Abbreviations: CNS—central nervous system; IL—interleukin; IFN—interferon; Th—T helper; TNF—tumor necrosis factor.

Sterile chronic low-grade inflammation also occurs in obesity [84], and it affects cognitive performance by compromising BBB integrity and neuronal circuits, and by activating immune cells [85]. Adipose tissue in the lean state contains multiple immune cells which operate in the Th2 state, including homeostatic ant-inflammatory macrophages, Treg cells, ILC2, invariant natural killer T (iNKT) cells, NK cells, and eosinophils. In obesity, this immune profile shifts towards a pro-inflammatory state, hallmarked by proliferation and recruitment of neutrophils, inflammatory macrophages, B cells, CD8 + T cells, Th1, and Th17 cells, along with a reduced abundance of eosinophils, Treg cells, iNKT cells and ILC2s [86]. Obesity-induced changes to the adipose tissue immune system impair local and systemic inflammation and metabolic health (Figure 1) [84]. The topic will be discussed later in detail.

### 2.4. Immunomodulatory Treatment in Neuropsychiatric Disorders

Immunomodulatory drugs have been long used for the treatment of neurological autoimmune disorders. Standard treatment for acute exacerbations includes steroids, intravenous immunoglobulin, cyclophosphamide, immunosuppressants, and monoclonal antibodies [1]. Many of those treatments had been previously tested in MS. Based on the evidence discussed, the immunomodulatory treatment of europsychiatric disorders has been considered with various degrees of success. For instance, monoclonal antibody treatments have been investigated in schizophrenia. Recent large-size RCT with recombinant humanized anti-human IL-6R monoclonal antibody tocilizumab resulted in a significant reduction in negative symptom score of the PANSS in schizophrenic individuals, although various adverse events were observed [87]. The administration of canakinumab, an IL-1β blocker, also showed a substantial decrease in the positive subscale scores of the PNASS and the hsCRP levels in individuals with chronic schizophrenia, without any adverse events reported [88]. Clinical trials are still ongoing with other monoclonal antibodies, such as natalizumab, an MS drug that attenuates neuroinflammation by inhibiting α4-integrin (NCT03093064) or siltuximab, an anti-IL-6 chimeric antibody used for Castleman disease (NCT02796859). Recently, anti-inflammatory therapeutic strategies, such as high-dose intravenous immunoglobulin were also applied to treat children with ASD, showing beneficial effects in subgroups with inflammation [89,90]. Similarly, accumulating evidence supporting the concept that immune dysregulation underlies depression, including anti-inflammatory effects of various antidepressants such as selective serotonin reuptake inhibitors [91], overall positive effects of non-steroidal anti-inflammatory drugs [92] or microglia inhibitor minocycline [93] led to testing other immunomodulatory treatments, such as methotrexate [94] or the TNF antagonist infliximab, showing positive results in subjects with drug-resistant depression [95]. Preclinical trials also demonstrated that TNF inhibitors could effectively alleviate amyloid pathology and tau phosphorylation [96], while clinical studies demonstrated the potential of etanercept, another TNF inhibitor, to improve language performance in a small cohort of AD subjects, although its full therapeutic effect remains to be validated [97,98]. Intraperitoneal administration of an IL-1R-blocking antibody also showed some potential to improve cognition and reduce tau pathology in mouse models of AD, but further clinical trials are needed in humans [99]. A potential protective role of non-steroidal anti-inflammatory drugs (NSAIDs) against AD has also been proposed [100], although the efficacy of these drugs is still debated.

## 3. Obesity and Neuropsychiatric Disorders

**Figure 2:**
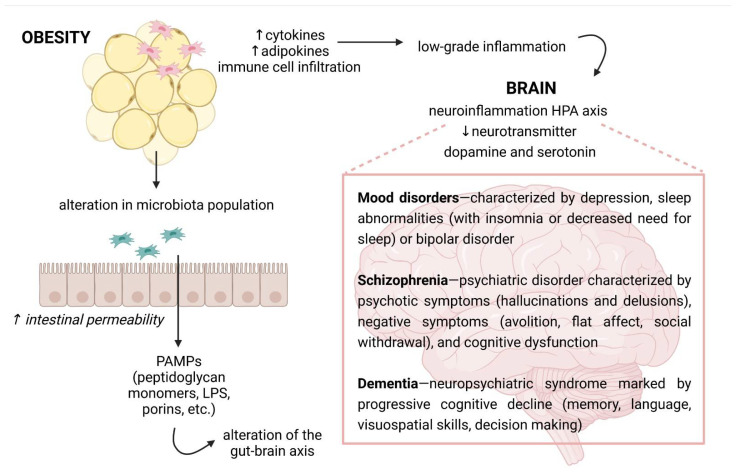
The possible links between obesity and neurological disorders. Because of a high secretion of cytokines and adipokines by the adipose tissue where immune cells infiltrate, obesity is recognized by chronic low-grade inflammation. Overnutrition is also linked with changes in gut microbiota composition and increased intestinal permeability. Then, it leads the activation of an immune response through microbial or pathogen-associated molecular patterns, e.g., pathogen-associated molecular patterns (PAMPs), such as lipopolysaccharide (LPS). The inflammatory molecules can reach the circulation and the central nervous system, where they may cause neuroinflammation. In turn, the immune activation in the brain can influence the hypothalamus–pituitary–adrenal (HPA) axis and neurotransmitter signaling, affecting cognition and behavior. Own drawing, based on [116].

Accumulating evidence has linked obesity to a wide spectrum of neurologic disorders [101,102,103]. However, it is important to recognize that obesity drives various additional processes, such as insulin resistance, which are also strongly linked to neurological deficits [104]. Numerous studies have demonstrated that obesity adversely affects the CNS and cognitive function (Figure 2). For instance, one meta-analysis showed a strong association between obesity and various neuropsychiatric disorders, including dementia and AD [105,106]. Data suggests that obesity doubles the risk of AD compared to individuals with normal weight [107], and that a higher body mass index (BMI) in midlife predicts increased risk of dementia in later life [106]. The results of a post-mortem study showed that elderly, morbidly obese individuals had higher levels of AD markers such as β-amyloid and β-amyloid precursor protein tau [108]. Several prospective studies demonstrated the link between obesity and an elevated risk of mild cognitive impairment [101,102,103], which usually proceeds dementia. Higher BMI has been associated with attention deficits, reduced verbal learning and memory, poor executive function and impaired decision-making. Neurological deficits in AD and mild cognitive impairment most likely result from structural changes in the brain and decreased cerebral integrity and volume, particularly in the hippocampus [109,110]. CT analysis demonstrated that women with atrophy of temporal lobe, area containing the hippocampus, had higher BMI, while longitudinal analysis of the same cohort showed that BMI was a good predictor for temporal lobe atrophy [109]. Other MRI studies confirmed the relationship [111,112]. The hippocampus and prefrontal cortex, which are particularly sensitive to obesity-associated changes [113], are essential for learning and memory, and reduction in hippocampal volume correlates with declines in memory performance [114]. Increased hippocampal atrophy in individuals with mild cognitive impairment has been shown to predict the development of AD [115].

## 4. Mechanisms Responsible for Obesity-Mediated Cognitive Impairment

In line with clinical evidence, studies on animal models of high-fat diet (HFD)-induced obesity demonstrate altered hippocampal structure and function that correlate with learning and memory deficits and impaired executive functions [117]. LTP is impaired in HFD-fed animals [118,119]. Furthermore, HFD results in decreased expression of neurogenesis markers, synaptic plasticity, and neuronal growth, including the brain-derived neurotrophic factor (BDNF) [120]. Increased levels of circulating pro-inflammatory cytokines/adipokines and free fatty acids (FFA) result in an acute response, an early hypothalamic inflammation manifesting mainly in increased expression of TNF-α and microglial and astrocytes activation [121]. The hypothalamus is crucial in maintaining energy homeostasis by regulating food intake and energy expenditure via neuroendocrine and autonomic signaling [122], and current data implies that the hypothalamic inflammation might be an early step in CNS dysfunction leading ultimately to cognitive abnormalities [112]. Microglia have particularly been implicated as the sensor of diet-induced hypothalamic inflammation due to the expression of Toll-like receptor (TLR)-4, capable of recognizing long-chain fatty acids (LCFAs) [123]. In animal models of obesity, activation of the TLR4 signaling pathway induces the production of pro-inflammatory cytokines (IL-1β, IL-6, and TNF-α [124]), activation of endoplasmic reticulum (ER) stress with subsequent activation of c-Jun N-terminal kinase (Jnk)/inhibitor of κB kinase-β/nuclear factor-κB (IKKβ/NF-κB) pathways, promoting the inflammatory signaling and gliosis in the hypothalamus [123,125]. Animal data shows that the expression of inflammatory markers in the hypothalamus, the step that proceeds any signs of inflammation in adipose tissue or peripheral blood, is induced just after a single day of HFD feeding [112]. An uncontrolled entry of pro-inflammatory mediators into the hippocampus amplifies neuroinflammation and promotes neurodegeneration [126]. Loss of the BBB and markers of hippocampal inflammation, including microglial activation, occur by 20 weeks of age in HFD-fed animals [127]. Increasing BBB permeability and continuous entry of pro-inflammatory mediators, immune cells, FFA, and triglycerides during obesity further amplify HFD-induced hippocampal injury and subsequent atrophy [117].

## 5. Obesity and the Gut Microbiota

Another factor contributing to obesity-mediated cognitive impairment involves gut microbes, which affect different aspects of human physiology, including maturation and function of the immune system [128,129]. Given the immunomodulatory properties of the gut microbiota, immune cell pathways within and peripheral to the CNS have been implicated as important mechanisms modulating a brain development, function and behavior [130,131]. It should not be a surprise, considering the density and dynamics of interactions at this interface. The gut microbiome harbors more than 22 million genes encoding various factors [132], ~70% of the body’s immune cells concentrate around the intestine [133], along with dense innervation around the intestine by neurons that are located entirely within intestinal tissue (108 intrinsic neurons) as well as neurons connecting the intestine to the CNS [134]. Gut microbiota is a dynamic ecosystem that changes both in composition and function throughout the lifespan in response to host factors, such as age and genetics [135], and to environmental factors, including stress, diet [136], drugs [137], etc.

Multiple animal studies have demonstrated an immediate and significant effect of diet on gut microbiota composition and function that affects essential host-microbe interactions [138]. Specific microbial communities and metabolites associated with lean body composition have been identified, and dysbiosis has been associated with obesity [139,140]. Animal studies have demonstrated that gut microbiota could promote adiposity and weight gain by altering the host gene expression, metabolic and inflammatory pathways as well as the gut–brain axis [141,142]. Various weight loss intervention studies in animals and humans have shown changes in microbial profile following the treatment and been implicated in the reduced weight and improved metabolic function [143,144]. Moreover, fecal microbiota transfer (FMT) has been demonstrated to recapitulate the functional metabolic phenotype observed in donors [145,146]. Furthermore, FMT in GF mice, using microbiota from obese individuals, leads to increased fat mass, and the inverse has also been observed. The observation that FMT from gastric bypass-operated mice to non-operated GF mice successfully transferred a lean, reduced fat mass phenotype provides further support for a causal relationship between gut microbiota and specific metabolic outcomes [145]. The study implies that bariatric surgery alters the microbiota composition and thus the composition of SCFAs produced, which in turn changes the host metabolism, GI hormone secretion, and insulin sensitivity. Similar results have been observed after bariatric surgery in humans [147,148]. The surgery shows to reduce the adiposity, hormonal and inflammatory statuses, improve insulin sensitivity as well as increase the bile acid pool and microbiota composition diversity. The overall abundance of Gammaproteobacteria and Verrucomicrobia (*Akkermansia*) is increased, while the abundance of Firmicutes is decreased [147].

**Figure 3:**
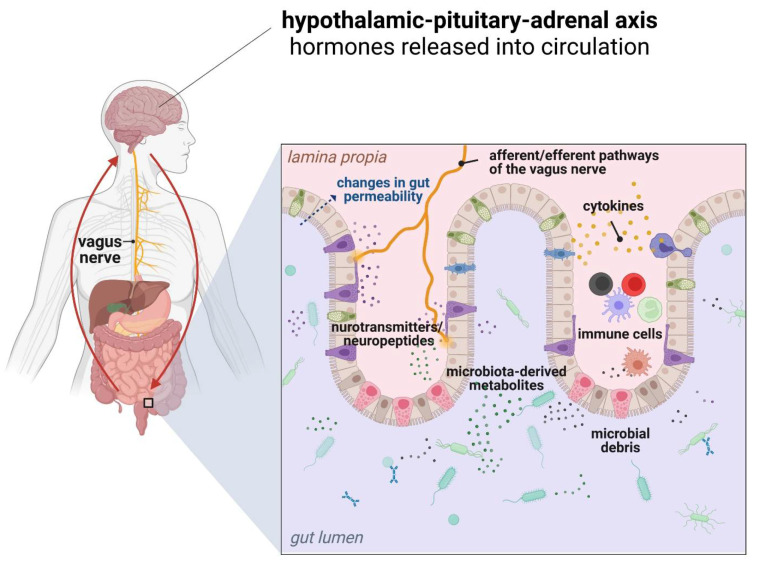
Various bidirectional communication pathways within the gut microbiota-immune system-brain axis [own drawing].

Alterations of gut microbiota composition and signaling pathways can affect brain function and behavior via several mechanisms. Activation of the innate immune response following exposure to microbial factors requires the recognition of microbe-associated molecular patterns (MAMPs) via pattern recognition receptors (PRR), including TLRs and NOD-like receptors (NLRs), which are expressed both in neurons and glial cells [149,150]. Since microbiota-derived TLR and NLR ligands can be found systemically during chronic inflammation, they can directly activate innate immune pathways to affect CNS function. For instance, LPS has been used to activate the immune system in models of schizophrenia, depression, and ASD in mice [151,152,153]. Different microbiota metabolites can affect brain function directly with potential targets including neurons, glia, astrocytes, oligodendrocytes, and endothelial cells or indirectly via activation of the vagus nerve, stimulation of endocrine cells (including enterochromaffin cells), and immune-mediated signaling (Figure 3) [129,154].

For instance, SCFAs, a microbial product of dietary fiber fermentation, play several roles, and their alterations have been associated with behavioral disorders, including depression [155], neurodevelopment [156], or anorexia nervosa [157]. SCFAs, including acetate, butyrate, and propionate, are a rich energy source for colonic epithelial cells, while the remaining pool enters the circulation where they can subsequently affect neurological function and development [154]. SCFAs regulate BBB permeability and microglia function and ultimately modulate host neuroinflammatory response [158]. SCFAs are believed to cross the BBB and directly influence the brain cells [159,160]. They also interact closely with the immune system via activation of G-protein-coupled receptors (GPCRs) and inhibition of histone deacetylases (HDAC) activity. A high-fiber diet, providing the higher concentrations of SCFAs, is associated with lower concentrations of circulating pro-inflammatory cytokines [161]. Activation of GPCRs (FFA2 and GPR109a) by SCFAs inhibits the inflammatory signaling pathways, and HDAC inhibition by SCFAs, particularly butyrate, results in a decreased inflammation in vivo [162]. SCFAs can also induce the production and release of enteroendocrine cells (EECs)-derived hormones, glucagon-like peptide 1 (GLP1), and peptide YY (PYY). These hormones indirectly modulate CNS-driven effects on postprandial satiety and emotional states, both of which are dysregulated in inflammation-associated diabetes and obesity [163].

Many microbial species can also modify primary bile acids [164], changing their signaling on the membrane and nuclear receptors, and altering their solubility and circulation levels [165]. Regulation of the presence and clearance of bile acids is involved in proper brain function, as alterations in these pathways lead to various neurological phenotypes, such as demyelination, motor dysfunction, neuroinflammation, seizures, and learning impairment [154]. Altered bacterial-associated bile acid levels have been observed in MS [154], AZ [166], PD [167], ASD [168], etc. Similarly, gut microbiota can influence levels of various hormones, particularly androgens and estrogens, by shifting the ratio of active and inactive steroid levels via various degradation and activation pathways [169]. Steroid hormone signaling is crucial for proper brain structure development, cognition, memory, decision-making, and sexual behaviors, and plays protective roles against social isolation and depression-like phenotypes [170,171,172]. Up to 15% of some of these hormones produced daily are detectable in the gut. It is believed that other gut microbiota metabolites, including proteinaceous toxins and amino acids, polyphenols, vitamins, lipids, etc., can also affect the brain cells directly, or indirectly via various pathways [154].

It has also been shown that gut microbiota could modulate the production of various neurotransmitters and synthesize neurotransmitters *de novo*. Thereupon, it can modulate the bioavailability and collaborate with the nervous system in the regulation of distinct functions [173,174]. For instance, Bacteroides ssp, Parabacteroides, and Escherichia species produce a major inhibitory neurotransmitter GABA, required for other bacteria, including Pseudomonas, Acinetobacter, and Mycobacterium genera [175]. Furthermore, the relative abundance of GABA-producing bacteria correlates with traits associated with depression and anxiety [175]. The abundance of GABA-producing bacteria has been negatively correlated with depression levels in patients [175], and abnormalities and the glutamate/GABA circuits in the brain have been hypothesized to be essential in anxiety disorders, MDD, BD, schizophrenia, and ASD [176,177,178]. Up to 90% of the host’s serotonin (5-HT) has also been found to derive from the gastrointestinal (GI) tract, implying the intestinal environment in modulating its synthesis [179]. In particular, spore-forming bacteria seem capable to modulate endocrine enterochromaffin cells in the gut lining to produce serotonin [180]. Moreover, metabolites upregulated by spore-forming bacteria or SCFAs could directly promote the production of serotonin and expression of the serotonin biosynthetic enzyme Tph1 in enterochromaffin cells [173,180]. GF mice show 2.8-fold lower plasma levels of serotonin compared to conventional mice [180]. Improved results in mouse models related to depression were obtained by probiotic treatment with Bifidobacterium spp. In this study, increased levels of serotonin in the brain and serotonin precursor in enterochromaffin cells have been observed [181]. Gut-derived tryptophan metabolites, which is a serotonin precursor, are another example of microbiome-dependent signals that modulate inflammatory responses. In order to locally strengthen the intestinal barrier, tryptophan metabolites target the aryl hydrocarbon receptor [182]. By inducing IFN-I signaling in astrocytes, they also alleviate CNS inflammation [183].

Glutamate, produced by various bacteria-namely *Corynebacterium glutamicum*, *Brevibacterium lactofermentum*, *Brevibacterium flavum*, and *Lactobacillus plantarum*, is another important neurotransmitter system affected by microbes [184,185]. In turn, *Escherichia* *coli*, *Proteus vulgaris*, and *Bacillus subtilis* produce relatively high levels of dopamine and norepinephrine as growth factors. The precise systemic pathways that affect this production are not well understood [156]. Immune cells of both myeloid and lymphoid lineages express various neurotransmitter receptors and can respond to stimulation by altering their inflammatory status.

Gut microbes can communicate with the CNS via the vagus nerve. This nerve directly links the muscular and mucosal layers along the GI tract to the brainstem. Hence, it is a well-established signaling pathway affecting feeding, anxiety-like, depressive-like, and social behavior [186,187,188]. Gut microbes and probiotics affect these behaviors via activation of vagal neurons. Then, they modify downstream neurological activity, including altered BDNE, GABA, and oxytocin signaling in the brain [186,187,188]. For instance, treatment of wild-type mice with *Lactobacillus rhamnosus* increases center exploration in the open field test and decreases immobility time in the forced swim test, and this effect is eliminated by vagotomy [186]. Increased latency in the step-down test of anxiety induced by DDS colitis also depends on the vagus nerve [188]. Furthermore, treatment with Bifidobacterium longum during DDS colitis does not ameliorate impairments in the step-down test in vagotomized mice, identifying a critical role for afferent vagal stimulation in bacteria-mediated changes in behavior. Conversely, the CNS controls intestinal motility, the release of neurochemicals, and intestinal immunity via the vagal nerve. Specifically, vagal nerve stimulation was implied to attenuate inflammatory responses through acetylcholine, suggesting that the immunomodulatory effects of probiotic strains such as Bifidobacterium and Lactobacillus may depend on the vagus nerve [189]. Cholinergic vagal efferent fibers stimulate enteric neurons that inhibit macrophage secretion of pro-inflammatory cytokines IL-1β, IL-6, IL-18, and TNF-α [190]. Furthermore, the biochemical alterations in the brain can also result in altered GI physiology, for instance via the HPA axis, which regulates the release of glucocorticoids from the adrenal gland [163]. Hormones produced by the HPA axis in response to stress, affect various organs to allow the host to adapt to the environmental changes. Activation of the stress response can result in altered intestinal permeability, motility, and mucus production, altogether leading to changes in microbial composition [131,191]. Stress-induced dysbiosis can in turn trigger intestinal inflammation through the Th17 cell-dependent release of IL-17A, which contributes to feed-forward activation of the stress response [192]. The gut microbiome also participates in regulating the HPA axis during homeostasis because microbiota deficiency exacerbates HPA activity in response to a moderate stressor [163]. Altogether, these findings support the idea that microbiota–gut–brain communication not only involves microbial modulation of brain function but also the reverse, namely, that CNS-induced biochemical changes result in altered intestinal physiology, microbiota composition, and immune function [131]. It is also worth noting that gut microbiota is highly involved in immune system maturation, the process that starts from the colonization of the neonatal gut by maternal microorganisms [193]. For instance, it was demonstrated in GF mice that specific species of bacteria were involved in the maturation of innate and adaptive gut immune T cell responses [194,195]. It was also shown that intestinal microbiota induced IFN-γ secretion by NK cells which in turn stimulated LAMP1+ TRAIL+ astrocytes, a specific anti-inflammatory astrocyte population associated with CNS development [196].

Accumulating evidence supports the concept that changes in the composition of gut microbiota can impact cognitive function at multiple levels. For instance, the complete absence of intestinal microorganisms can induce various alterations in host cognition. GF mice exhibit an impaired ability to remember a familiar object when presented with a novel object, as well as impaired working memory in remembering a familiar environment in the spontaneous alteration task [197,198]. They also present altered hippocampal BDNF expression [196], which plays an important role in synaptic plasticity and cognition [199], suggesting a crucial involvement of microbes in regulating hippocampal-dependent memory function. The use of antibiotics disrupting the microbial composition can also have a detrimental effect on brain function and behavior. Antibiotic administration at an early age has been demonstrated to induce gut microbiota changes, along with the subsequent object recognition memory impairments and altered hippocampal BDNF expression when measured during adulthood [200]. Similar memory deficits associated with altered hippocampal expression of signaling molecules relevant to cognition, including BDNF, glutamate ionotropic receptor NMDA type subunit 2B (GRIN2B), serotonin (5-HT) transporter, and neuropeptide Y (NPY), following antibiotic administration have been observed in adult subjects in other studies [201,202]. Multiple animal studies have demonstrated the beneficial effects of probiotic and prebiotic treatments in modulating health and preventing or restoring cognitive deficits associated with dysbiosis [197,203,204]. Altogether, the data implies the importance of the gut microbiota in memory performance and hippocampal function.

However, there is only a limited number of studies supporting the role of gut microbiota in modulating human cognition. For instance, the microbiota composition of obese and non-obese individuals was associated with results in speed, attention, and flexibility in a Trail Making Test coupled with alterations in neural activity in the thalamus, hypothalamus, and amygdala, implying that obesity affects the microbiota composition and subsequent cognitive function [205]. Another study showed that higher levels of Bacteroides in one-year-old babies were associated with higher cognitive performance tested with the Mullen Scales of Early Learning [206]. This group was also less likely to be born via C-section, which is consistent with the results of other studies linking mode of delivery with child cognitive development [207]. Treatment with Lactobacillus strains in healthy elders shows improved cognitive test performance compared with a placebo group [208]. In healthy women, consumption of a fermented milk product supplemented with a probiotic modulates the activity in brain regions involved in cognitive performance during an emotional attention test [209]. Improvements in memory tasks and subjective improvements in mood are observed in healthy subjects treated with prebiotic inulin [210]. Probiotic administration is shown to alter brain activities related to emotional memory, decision-making tasks, anxiety, and negative affect, which is affected by shifts in the gut microbiome [211,212].

Various intrinsic and extrinsic factors can trigger an imbalance in the interplay between gut microbiota and the brain [213,214]. In this regard, biopsychosocial disorders and functional GI conditions have been linked to dysbiosis [213]. For instance, irritable bowel syndrome (IBS) is frequently accompanied by anxiety, depression, and obsessive-compulsive disorder [214,215], as well as schizophrenia and panic disorder [215], while the signs and symptoms include abdominal pain and altered intestinal motility or secretion, all controlled by the brain [215,216,217]. Probiotic *Bifidobacterium Longum* administration has been shown to reduce depression and alter brain activity in IBS individuals [218].

Gut dysbiosis has been also implicated in the pathogenesis of a growing list of neurological and psychiatric disorders [163]. For instance, although the symptoms of ASD are heterogeneous, they are characterized by altered behavior, including social communication, social interactions, and repetitive behaviors [219]. About 70% of ASD children report comorbid GI disturbances, such as bloating, constipation, and diarrhea, which correlate with the behavioral severity, implying the gut–brain link in neurodevelopment [220,221]. ASD children exhibit increased susceptibility to intestinal inflammation [222] and altered permeability [223]. The significant improvement in behavioral symptoms observed in children with ASD treated with broad-spectrum antibiotic vancomycin suggests that the gut microbiota may contribute to the disorder [224,225]. Multiple studies have found alterations in the composition of the gut microbiota and their metabolites in ASD children, including the reduced abundance of the beneficial bacteria genus Bifidobacterium, along with the increased abundance of potentially pathogenic Desulfovibrio and *Clostridia genera* [226,227]. Some of these significant changes show potential for early diagnosis of ASD [228]. Although limited in number, studies targeting the gut microbiota conducted so far show beneficial effects of probiotic, prebiotic, and FMT for ASD subjects [229,230]. The treatments reduce GI symptoms and improve ASD core symptoms, including social skill deficits and repetitive behavior.

There is also growing evidence of a role for the microbiota–brain axis in MDD [231,232], although most of the studies have been conducted in preclinical models [233]. A transfer of fecal microbiota collected from MDD subjects to rats resulted in depressive and anxious phenotypes, which has not been observed with FMT collected from healthy humans [234]. Alterations in microbial composition in MDD subjects have been found in multiple studies, however, the results are not consistent, suggesting further research is needed [235,236,237]. A recent meta-analysis shows a reduced abundance of *Coprococcus*, *Faecalibacterium*, *Ruminococcus*, *Bifidobacterium*, and *Escherichia*, and an increased abundance of Paraprevotella in MMD subjects [238]. Similar results came from a study of the microbiota composition of >1000 individuals enrolled in Belgium’s Flemish Gut Flora Project, which clustered individuals into four enterotypes based on their microbiome composition [239]. Individuals with depression or lower quality of life scores were over-represented in an enterotype characterized by a lower level of genus *Faecalibacterium*, in addition to overall reduced microbial diversity. Further analysis showed that higher quality of life indicators was associated with higher levels of bacteria producing SCFA butyrate, including *Faecalibacterium* and *Coprococcus*. MDD has been also associated with physiological changes, including the alterations in gut permeability and systemic inflammation manifested with increased levels of IL-1β, IL-6, TNF-α, and CRP [240]. Numerous studies focused on probiotic intervention, mostly using various strains of Lactobacillus and Bifidobacterium, have demonstrated improvements to mood ratings in the elderly [241,242], improvements in depression scores [243,244], and improved cognitive functions [245] in MDD subjects, significantly lower levels of anxiety and postnatal depression after pregnancy [246]. Other interventional attempts have failed to show any benefits of probiotic administration [208,247]. It is also worth noting, that poor diet is currently an accepted risk factor for depression, as well as a therapeutic target [248].

Evidence also suggests a role of the GI immune response in the pathogenesis of schizophrenia, where risk factors include *Toxoplasma gondii* infections, food intolerance, GI inflammation, and altered intestinal permeability [249,250,251]. A recent metagenomic analysis of the oropharyngeal microbiota in schizophrenic and healthy individuals identified significant differences in *Proteobacteria*, *Firmicutes*, *Bacteroidetes* and *Actinobacteria* among both groups, with the fungi Ascomycota present in great abundance in schizophrenic individuals [252]. Results of other studies are inconsistent, but generally report a decreased diversity of gut microbiota and increased abundance of the *phylum Proteobacteria* [253]. One recent study linked an abundance of *Succinvibrio* and *Coryneobacterium* with the severity of disease symptoms, which might a diagnostic potential [254]. FMT in GF mice reduces hippocampal glutamate, and increases levels of glutamine and GABA, suggesting that the schizophrenic microbiome itself can affect neurochemistry [255]. A small number of human studies have been conducted so far focused on the therapeutic effect of probiotic intervention in schizophrenia, however with limited success [256,257].

Another serious neuropsychiatric illness, BD, has been also linked to dysbiosis [258]. Implications for the microbiome involvement emerged with the observation that BD subjects are twice as likely as other subjects to be recently treated with systemic antibiotics [259]. Fecal microbiome analysis of BD subjects shows a reduced abundance of *Bacteroides* and *Firmicutes*, particularly *Faecalibacterium*, and overall reduced diversity of gut microbiota [260]. Preliminary results from clinical trials indicate that microbiome-targeted therapies might be useful in the treatment or even prevention of BD [261].

Growing evidence also suggests an important role of the gut–brain axis in the etiopathogenesis of PD, AD and dementia, MS, and epilepsy. Altered microbiota composition has been linked to PD in multiple studies [262,263,264]. GI symptoms, primarily in the form of constipation, can occur in up to 80% of PD subjects and can precede disease diagnosis by many years [265]. An appendectomy has been recently considered a potential prophylactic procedure for PD initiation [266]. Preclinical studies provide further evidence showing that FMT from PD subjects in GF mice results in hallmarks of the disease, namely motor deficits, and neuroinflammation, accompanied by the overexpression of protein α-synuclein, while the treatment with antibiotics improves the symptoms [267]. Intestinal α-synuclein aggregates and then propagates from the gut to the brain via the vagus nerve [267,268], and its aggregation seems to be modulated by LPS [269]. Activated microglia subsequently recruit enteric α-synuclein-specific T cells to the substantia nigra, where they promote further neuroinflammation [267]. Intestinal inflammation itself can also lead to CD8+ T cell infiltration and alter dopaminergic pathways in the brain [270]. Studies on gut microbiota in PD show significant changes in microbial composition, such as a decreased abundance of *Provotellacae*, increased abundance of *Enterobacteriaceae* [271], or increased abundance of Bacteroidetes, Proteobacteria, and Verrucomicrobia at the phylum level [272], compared to healthy controls. Studies using metagenomic shotgun analysis techniques show the increased abundance of *Verrucomicrobiaceae* and *Firmicutes* and decreased abundance of *Prevotellaceae* and *Erysipelotrichaceae* in PD subjects [262]. The role of dysbiosis in the etiopathogenesis of PD is further supported by the beneficial effects of probiotic interventions and FMT [273,274,275,276].

Similarly, there is growing evidence supporting the concept of microbial origin and progression of AD and dementia [166,277]. In general, AD subjects show a reduced abundance of Eubacterium, but a higher abundance of *Escherichia* and *Shigella* [278], along with changes in *Bacteroides*, *Actinobacteria*, *Ruminococcus*, *Lachnospiraceae*, and *Selenomonadales* [279]. Other studies show a reduced proportion of phylum Firmicutes, but enriched Proteobacteria in AD subjects with amnestic mild cognitive impairment [279,280]. Experimental evidence [281] supports previous research identifying the amyloid protein acting as a possible antimicrobial peptide in the brain [282]. Interestingly, amyloid-like proteins can be produced by bacteria and have been shown to increase α-synuclein pathology in aged rats, suggesting that molecular mimicry might be involved [283]. It is worth noting, however, that although the accumulation of Aβ peptide and abnormal forms of tau protein are traditionally regarded as the indicators of AD pathology, it does not necessarily infer causality. Results of clinical trials targeting microbiome in AD are not consistent, and a recent meta-analysis does not confirm the beneficial effects of probiotic supplementation with Lactobacillus and Bifidobacterium strains [284].

MS is a CNS-related autoimmune disorder defined by accruing motor deficits, blurred vision, and changes in sensibility that appear spontaneously with little to no prodromal signals [285]. Based on the immunoetiology of MS, much interest is now [275,276] focused on possible therapeutic intervention via the gut microbiota [286,287]. Fecal transfer from MS subjects to mice leads to autoimmune encephalomyelitis, which is one of the hallmarks of MS [288]. GF mice also exhibit resistance to developing experimental autoimmune encephalomyelitis in a mouse model of MS, which was promoted by gram-positive segmented filamentous bacteria in the gut [286,288]. Interestingly, the symptoms correlate with an increased presence of Akkermansia. The observation fits with a recent study demonstrating the increased abundance of *Akkermansia muciniphila* and *Acinetobacter calcoaceticus* in fecal samples of MS subjects [289]. In addition, MS subjects also show reduced levels of *Parabacteroides distasonis*, a species associated with anti-inflammatory effects.

## 6. Maternal Immune Activation and Neuropsychiatric Disorders

**Figure 4:**
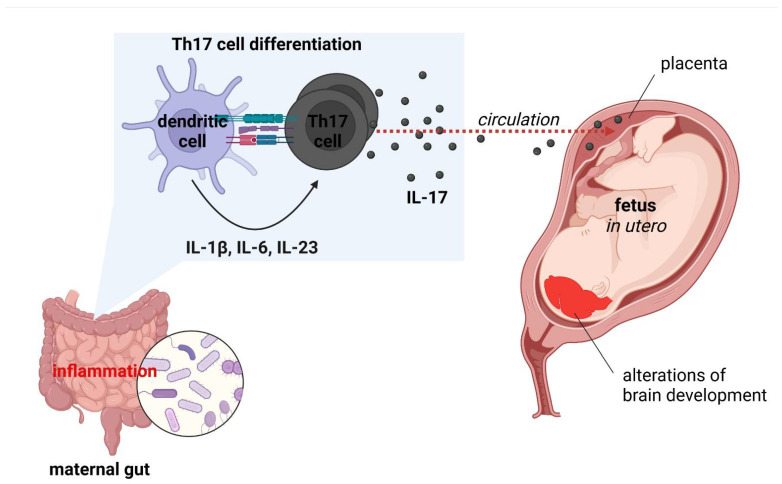
Maternal immune activation (MIA) model of autism spectrum disorder (ASD) development [own drawing]. Abbreviations: IL—interleukin; Th—T helper.

Accumulating evidence indicates that overactivation of the immune system increases brain vulnerability, heightening the likelihood of psychiatric symptoms [290]. At the epidemiological level, the hypothesis is supported by a connection between psychiatric and systemic autoimmune disorders [291,292]. Both psychiatric [293] and autoimmune disorders [294] also manifest more frequently in women, whose immune system is in general overactive, which has evolutionary bases. Various studies demonstrate that, e.g., schizophrenia, depression, suicidal ideation and post-traumatic stress disorder are characterized with increased levels of inflammatory markers, particularly IL-1β, IL-2R, IL-6, IL-17A, TNF-α and CRP [1]. Several studies have indicated that link between serum levels of inflammatory markers with disease activity and a negative correlation with cognitive performance [1]. Abnormal changes in the innate immune system within CNS may arise from psychiatric disorders, such as genetic defects in microglial signaling pathways. They entail abnormal development of brain circuits and neuropsychiatric symptoms, similar to the case of hereditary diffuse leukoencephalopathy with spheroids [295]. The nervous system is highly vulnerable to environmental factors during development. The excessive inflammatory conditions, recognized as maternal immune activation (MIA), mainly higher levels of IL-6 and IL-17A, causing to the pathology of neurodevelopmental disorders [4]. Abnormal immune activity during development has been shown to alter the microglia development. Thereupon, it can disrupt the neurodevelopmental processes that microglia typically manage, leading to neurological dysfunction [296]. Maternal inflammation and stress have also been demonstrated to alter the vaginal microbiome in pregnant mice. Consequently, the developmental process in the offspring is disrupted [297,298]. Numerous epidemiological studies have reported maternal infection during pregnancy with a higher risk for neurodevelopmental disorders [299,300,301]. Other maternal risk factors for MIA, mainly autoimmune diseases, obesity, diabetes, and genetic risk variants are linked to offspring neurological dysfunction [302]. Furthermore, the cumulative data indicate that MIA is a significant risk factor for neurodevelopmental disorders (ASD, schizophrenia, cerebral palsy, epilepsy, neurodegenerative diseases, behavioral abnormalities, etc.) [4,303]. In mouse models of ASD, maternal infection or immune activation during pregnancy induce DC-dependent differentiation of IL-17-producing Th17 cells in the gut [304]. Systemic IL-17 crosses the placental barrier and alters the developing CNS, resulting in brain and behavioral endophenotypes of autism in the offspring. These observations imply that intestinal inflammation can stimulate neurological abnormalities through the humoral transit of inflammatory factors across the feto-maternal interface (Figure 4). Importantly, evidence suggests that MIA could ‘prime’ microglia and sensitize cells to subsequent inflammatory stimuli [305]. Disruption of microglial function later in life can be triggered by various environmental and psychological factors, including obesity-driven inflammation.

## 7. Conclusions

**Figure 5:**
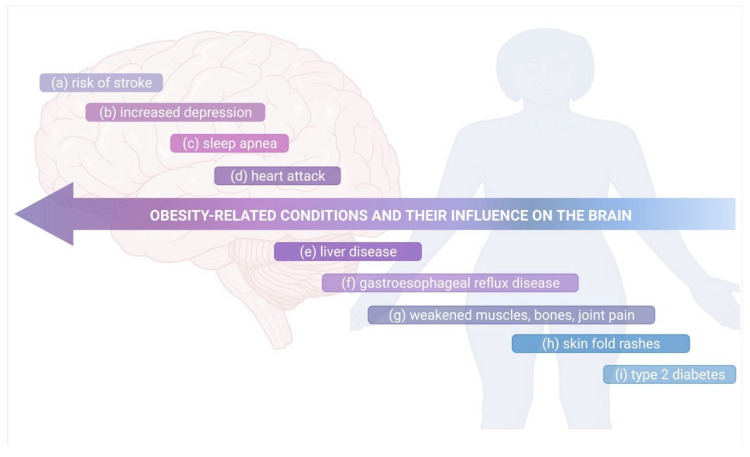
The obesity-related conditions and their influence on the brain. Obesity can: (**a**) lead to high blood pressure, causing stroke; (**b**) influence the mental health, increasing depression and issues of body image; (**c**) cause airways to small, leading to sleep apnea, where the breathing stops for periods of the time at night; (**d**) harden the arteries, increasing thereupon the risk of heart attack due to high blood pressure, cholesterol and blood sugar occurring in this disorder; (**e**) built up excess fat around the liver, leading to damage and its failure; (**f**) induce a higher risk of GERD, where stomach acid leak into your esophagus; (**g**) muscle mass and bone density to deteriorate, leading to disability and fracture risk, it can put a strain on your joints, causing pain and stiffness; (**h**) lead to discolored and thickened rashes that occur when the skin folds; (**i**) make the human body resistant to insulin, leading to high blood sugar, and increase the risk of type 2 diabetes. Own drawing, based on [316].

Current evidence indicates that innate and adaptive immunity can have an influence on neurodevelopment and CNS homeostasis, however, any immune dysregulation can disturb the fragile equilibrium between immune cells, neurons, and glial cells. As a result, harmful effects refer to neurological symptoms and also to higher-order network function. Increased visceral adiposity has been considered as a main reason for the development of a wide range of neuropsychiatric conditions [117]. The precise mechanisms of obesity-induced neurological dysfunctions are not clear, however accumulating data suggests the essential role of obesity-driven inflammation and gut dysbiosis [306,307], although other mechanisms might also contribute to it, such as metabolic dysfunction, oxidative stress, and ER stress [308,309]. A Western diet (WD) relies on the consumption of high-saturated fat and sugar, resulting in changes in the expression of intestinal barrier markers [310]. Moreover, the diet decreases EEC-derived GLP1, induces mediobasal hypothalamic inflammation through microglia-release of IL-1β, IL-6, and TNF-α, and it also triggers central resistance to the leptin (appetite-regulating hormone). WD may induce addictive-like eating behaviors or is even associated with the occurrence of increased anxiety-like behaviors [140,311,312]. Hypothalamic inflammation can be attenuated in the absence of a gut microbiome, and behavioral abnormalities can be conferred through FMT from HFD-treated mice to naïve mice, suggesting that diet-induced dysbiosis of the gut microbiome contributes to the CNS phenotypes [140,312]. The dietary intervention combined with physical activity has been demonstrated to effectively reduce obesity and restore neurological function. It can not only lower the systemic inflammation and improve the metabolic profile, but also improve both nerve and cognitive functions, at least in some individuals with this heterogenic condition [313]. Recent research has shown that the beneficial effects of physical exercises on cognitive function, which are tied to increased plasticity and reduced neuroinflammation within the hippocampus [314], result from the activity of complement cascade inhibitors including clusterin (CLU) [315]. Further research is needed to elucidate the link between obesity, inflammation, gut microbiota, and neuropsychiatric disorders. Figure 5 summarizes the relation between the obesity-related conditions and their influence on the brain that is commonly recognized.

## Data Availability

Not applicable.
